# Structural brain variability in recent-onset and chronic schizophrenia: evidence from person-based similarity index analysis

**DOI:** 10.1017/neu.2025.10043

**Published:** 2025-11-03

**Authors:** Young Tak Jo, Jungsun Lee, Sun Min Kim, Hyeongyu Park, Sung Woo Joo

**Affiliations:** 1 Department of Psychiatry, Kangdong Sacred Heart Hospital, Hallym University College of Medicine, Seoul, Korea; 2 Department of Psychiatry, Asan Medical Center, University of Ulsan College of Medicine, Seoul, Korea

**Keywords:** Schizophrenia, neuroimaging, cerebral cortex, psychiatric disorders, MRI

## Abstract

**Objective:**

Structural abnormalities in cortical and subcortical brain regions are consistently observed in schizophrenia; however, substantial inter-individual variability complicates identifying clear neurobiological biomarkers. The Person-Based Similarity Index (PBSI) quantifies individual structural variability; however, its applicability across schizophrenia stages remains unclear. This study aimed to compare cortical and subcortical structural variability in recent-onset and chronic schizophrenia and explore associations with clinical measures.

**Methods:**

Neuroimaging data from 41 patients with recent-onset schizophrenia, 32 with chronic schizophrenia, and 59 healthy controls were analysed. The PBSI scores were calculated for cortical thickness, surface area, cortical grey matter volume, and subcortical volumes. Group differences in PBSI scores were assessed using linear regression and analysis of variance. Correlations between the PBSI scores and clinical measures were also examined.

**Results:**

Both patients with recent-onset and chronic schizophrenia exhibited significantly lower PBSI scores than healthy controls, indicating greater morphometric heterogeneity. However, significant differences between the recent-onset and chronic patient groups were limited to subcortical and cortical thickness PBSI scores. Correlations between PBSI scores and clinical symptoms are sparse and primarily restricted to surface area variability and symptom severity in patients with recent-onset schizophrenia.

**Conclusion:**

Patients with schizophrenia show marked structural brain heterogeneity compared with healthy controls, which is detectable even in the early stages of the illness. Although there were few differences in PBSI scores between the recent-onset and chronic schizophrenia groups and limited correlations between PBSI scores and clinical measures, the PBSI may still provide valuable insights into individual differences contributing to clinical heterogeneity in schizophrenia.

## Significant outcomes

- Across all cortical and subcortical measures, PBSI scores were lower in schizophrenia than in healthy controls, indicating greater inter-individual morphometric heterogeneity.
- Differences between recent-onset and chronic schizophrenia were modest and confined to cortical thickness and subcortical PBSI.
- In recent‑onset schizophrenia, lower PBSI for surface area correlated with higher PANSS-positive, general, and total scores; no robust associations with cognitive measures were observed.

## Limitations

- The cross‑sectional, single‑centre design and consent‑based recruitment limit causal inference and may reduce generalisability to more severely affected populations.
- Antipsychotic exposure was not controlled for, so medication effects on morphometry cannot be excluded.
- Cognitive assessments were limited and non‑uniform across cohorts, and potential confounders – including intelligence, education, and socioeconomic status – were not controlled for.

## Introduction

Schizophrenia is a chronic, debilitating, psychiatric disorder characterised by hallucinations, delusions, and disorganised behaviour. It is widely recognised as a severe mental illness that imposes a substantial global disease burden (Charlson *et al*., 2018). Consequently, considerable effort has been devoted to identifying the neurobiological underpinnings of schizophrenia, particularly using neuroimaging techniques (Kraguljac *et al*., 2021; Howes *et al*., 2023). Numerous studies have consistently reported structural abnormalities, including reductions in cortical thickness and surface area across widespread regions of the cerebral cortex, although these effects are typically modest in magnitude (van Erp *et al*., 2018; Madre *et al*., 2020).

Although cortical abnormalities are well-documented, their clinical utility remains limited, likely because of substantial interindividual variability in morphometric features among patients with schizophrenia (Brugger & Howes, 2017). Most neuroimaging studies rely on group-level comparisons of brain morphometry (Thompson *et al*., 2020), and implicitly assume distinct patient and control populations. However, such approaches often overlook individual differences, limiting the exploration of the neural correlates underlying clinical heterogeneity. Indeed, studies have suggested that group-level cortical differences account for only a small proportion of the observed variance in schizophrenia (Wolfers *et al*., 2018; Wolfers *et al*., 2021). Thus, average-based case–control comparisons inadequately capture individual variability.

Recently, the Person-Based Similarity Index (PBSI) was introduced to assess variability in brain morphometric measures within diagnostic groups (Doucet *et al*., 2019). This index quantifies the similarity of an individual’s cortical and subcortical profiles relative to peers within the same diagnostic category (Doucet *et al*., 2020a; Doucet *et al*., 2020b; Antoniades *et al*., 2021), facilitating the investigation of inter-individual variability. Lower PBSI scores indicated greater heterogeneity among patients. Studies have consistently reported that patients with schizophrenia exhibit lower PBSI scores for cortical thickness than healthy controls. Additionally, subsets of patients with markedly deviant PBSI scores for sulcal width exhibit impaired cognitive performance (Doucet *et al*., 2020b; Janssen *et al*., 2021). Lower PBSI scores for cortical thickness, surface area, and subcortical volume have also been observed in patients with first-episode psychosis (Antoniades *et al*., 2021) and schizophrenia (Omlor *et al*., 2025) compared to healthy controls. Our previous work employing the PBSI similarly demonstrated that patients with schizophrenia display lower PBSI scores across all cortical measures – cortical thickness, surface area, cortical grey matter volume, and local gyrification index – indicating greater morphometric heterogeneity compared with controls (Joo *et al*., 2024).

However, most PBSI-based studies have treated patients with schizophrenia as a single group compared to healthy controls, potentially obscuring the differences between recent-onset and chronic patients. Some pathological features manifest early, occasionally preceding the onset of psychotic symptoms, whereas others progressively develop or worsen during the course of the illness. Several studies have documented distinct neurophysiological profiles in patients with chronic and recent-onset schizophrenia, including differences in intrinsic brain activity (Gong *et al*., 2020), mismatch negativity (Salisbury *et al*., 2018), brain metabolites (Natsubori *et al*., 2014), serum cytokines, and other neural markers (Cai *et al*., 2022). Cortical thinning and related structural changes are widespread and pronounced in patients with chronic schizophrenia (Ellison-Wright *et al*., 2008; Wannan *et al*., 2019), supporting the hypothesis of progressive structural deterioration (Zhao *et al*., 2022). Clinical differences, particularly in cognitive deficits, have also been reported between patients with recent-onset and chronic schizophrenia, although the findings remain inconsistent (McCutcheon *et al*., 2023).

Therefore, assessing cortical morphometric variability in relation to illness duration using PBSI can provide valuable insights. In the present study, we analysed neuroimaging data collected from a university-affiliated hospital to investigate the structural variability in cortical morphometry between patients with recent-onset and chronic schizophrenia and healthy controls. Specifically, our aim was to evaluate how structural variability in cortical and subcortical regions differs from that in healthy controls according to illness duration and to explore associations between structural deviations and clinical variables within each patient subgroup. Based on our previous findings, we hypothesised that structural variability in cortical and subcortical morphometry would be greater among patients with schizophrenia than among healthy controls and would significantly differ between recent-onset and chronic patient groups, reflecting distinct neurobiological trajectories linked to illness progression.

## Methods

### Study population

Study participants were drawn from three cohorts (AMC 1, AMC 2, and AMC 3) recruited from a single university-affiliated hospital. Patients with schizophrenia were categorised as recent-onset (≤ 5 years) and chronic (> 5 years) based on illness duration. The five-year threshold was selected in line with prior literature (Newton *et al*., 2018), which documented five years as a commonly used cut-off for operationalising recent-onset schizophrenia. Information on antipsychotic medication was available for AMC1 and AMC3 but not for AMC2. To allow comparability across different drugs, all recorded doses were standardised to olanzapine-equivalent doses. Psychiatric symptom severity was assessed using the Positive and Negative Syndrome Scale (PANSS) and the Global Assessment of Functioning (GAF). As cognitive assessment tools varied across cohorts, cognitive analyses were limited to measures that were directly comparable across cohorts. Specifically, the Full-Scale Intelligence Quotient (FSIQ) and the Memory Quotient (MQ) were obtained from AMC1 and AMC3; AMC2 administered the Cogstate Brief Battery only and therefore did not contribute to the FSIQ/MQ analyses. This study was conducted in accordance with the Declaration of Helsinki and approved by the Institutional Review Board (IRB) of the Asan Medical Center (IRB No. 2021-1128). The detailed inclusion and exclusion criteria, clinical assessment methodologies, and ethical considerations are described in the following sections. The scanner details and magnetic resonance imaging (MRI) acquisition parameters are provided in Supplementary Table 1.

### AMC 1

The participants in this cohort were recruited between 2012 and 2015. A psychiatrist diagnosed patients with schizophrenia according to the Diagnostic and Statistical Manual of Mental Disorders, Fourth Edition (DSM-IV). All patients experienced their first psychotic symptoms (e.g., delusions or hallucinations) within the preceding five years. In the control group, neither the participants nor their first-degree relatives had any Axis I psychiatric diagnoses based on the DSM-IV-TR criteria. All participants were right-handed and aged between 20 and 40 years. Exclusion criteria included any condition that could affect brain function or interfere with neuropsychological assessments or MRI procedures. After visual inspection of the MRI scans, the final sample comprised of 49 patients (19 males, 30 females) and 24 healthy controls (9 males, 15 females). Psychiatric symptoms and cognitive function were assessed within one week of MRI scanning. Psychiatric symptoms were evaluated using the PANSS, and cognitive function was measured using the age- and sex-adjusted short form of the Wechsler Adult Intelligence Scale-Third Edition (WAIS-III) and a video-based social cognition scale (VISC). Written informed consent was obtained from all participants and ethical approval was obtained from the IRB of Asan Medical Center (IRB File No. 2012-0485).

### AMC 2

The study participants in this cohort were recruited between August 2017 and February 2020. Although we initially included patients with both schizophrenia and bipolar disorder, the current analysis focused solely on patients with schizophrenia and healthy controls. Diagnoses were made using a Structured Clinical Interview for DSM Disorders (SCID). Controls were defined as individuals with no history of Axis I disorders or their first-degree relatives, as confirmed by SCID. All participants were between 20 and 55 years of age, right-handed, free from physical illnesses that might affect brain function, and had an FSIQ score > 80. After visual MRI inspection and exclusion of patients with bipolar disorder, the final sample comprised 27 patients (9 males, 18 females) with schizophrenia and 55 healthy controls (16 males, 39 females). Psychiatric symptoms and cognitive function were assessed within one week of MRI scanning. Psychiatric symptoms were evaluated using the PANSS, and cognitive functions were assessed using the Cogstate Brief Battery, which covers processing speed, attention, working memory, visual learning and memory, verbal learning and memory, executive function, and social cognition. All participants provided written informed consent and the IRB of Asan Medical Center approved the study (IRB File No. 2017-0839).

### AMC 3

The study participants in this cohort were recruited from June 2021 to December 2023. Diagnoses of schizophrenia were based on the Diagnostic and Statistical Manual of Mental Disorders, Fifth Edition (DSM-5). All participants were between 20 and 70 years of age, had a duration of illness exceeding five years, and were right-handed. The exclusion criteria included intellectual disability, substance abuse or dependence within the past six months, neurological disorders, head trauma causing unconsciousness longer than 3 min, and other unstable conditions potentially influencing brain function. Written informed consent was obtained from all participants prior to their participation in the study. Initially, 69 patients were enrolled, of whom 14 withdrew consent, resulting in 55 patients completing the study. Clinical assessments were conducted within one week prior to MRI scanning. Psychiatric symptoms were assessed using the PANSS and cognitive function was measured using the shorter version of the Wechsler Adult Intelligence Scale-Fourth Edition (WAIS-IV), Rey-Kim Memory Test, and Kims Frontal-Executive Neuropsychological Test. The study was conducted in accordance with the Declaration of Helsinki and approved by the IRB of Asan Medical Center (IRB No. 2021-1128, 2022-1193). After excluding two participants owing to poor MRI image quality and one participant with significant neurological abnormalities, the final sample comprised 52 patients (26 males, 26 females) with schizophrenia.

### Image processing

Image analysis was performed using the FreeSurfer automated pipeline (version 7.4; https://surfer.nmr.mgh.harvard.edu/fswiki/recon-all). Prior to analysis with FreeSurfer, all MRI scans underwent rigorous visual inspection for quality control. To capture fine topological details of cortical regions, we applied the Schaefer atlas consisting of 100 cortical parcellations (Schaefer *et al*., 2018)(https://github.com/ThomasYeoLab/CBIG). Specifically, we measured surface area, cortical thickness, and grey matter volume within these 100 cortical regions. Additionally, volumes were quantified in 14 subcortical regions: bilateral thalamus, caudate, putamen, pallidum, hippocampus, amygdala, and accumbens area. To harmonise data across cohorts, the ComBat harmonisation method was applied using group status as the sole covariate.

### PBSI computation for cortical and subcortical measures

We computed subject-level PBSI scores separately for each morphometric measure – cortical thickness, surface area, grey matter volume, and subcortical volumes – within each diagnostic group, following procedures validated in previous studies (Doucet *et al*., 2019; Doucet *et al*., 2020a; Doucet *et al*., 2020b; Antoniades *et al*., 2021; Janssen *et al*., 2021). For each cortical measure (cortical thickness, surface area, and grey matter volume), individual profiles were constructed by concatenating the 100 regional values; subcortical profiles were constructed by concatenating the 14 regional volumes. Spearman’s correlation coefficients (rho) were calculated between each participant’s profile and those of all other individuals within the same group, yielding n – 1 coefficients per participant (where n represents the group size). The average of these coefficients was used as the individual PBSI scores for each cortical or subcortical measure. In addition, we derived the PBSI Cortical score by aggregating the scores across all cortical measures, and the PBSI Total score by aggregating the scores across both cortical and subcortical measures. To account for scale differences, the values were converted into z-scores for each participant. Thus, the PBSI score quantifies the similarity of an individual’s cortical and subcortical structural profile to that of others within the same diagnostic group.

## Statistical analyses

To minimise confounding effects on PBSI scores, we used MatchIt to perform age- and sex-matching within the pool of 128 patients and 79 healthy controls, resulting in final group sizes of 41 patients with recent-onset schizophrenia (34 from AMC1, 7 from AMC2), 32 with chronic schizophrenia (11 from AMC2, 21 from AMC3), and 59 healthy controls (20 from AMC1, 39 from AMC2). Outliers identified using the 1.5 × interquartile range (IQR) criterion on boxplots were excluded from the analyses. Independent t-tests were applied when comparing two groups on continuous demographic and clinical variables, whereas analysis of variance (ANOVA) with Tukey’s post-hoc tests was used for comparisons across all three groups. Chi-square tests were applied to categorical variables. Group differences in PBSI scores were assessed using linear regression models adjusted for age and sex, followed by an ANOVA with Tukey’s post-hoc tests. Associations between the PBSI scores and clinical variables were evaluated using Pearson’s correlation coefficients. Multiple comparisons were corrected using the false discovery rate (FDR) method for all tests examining the associations between the PBSI scores and clinical measures within each participant group, accounting for seven comparisons. Statistical analyses were performed using R version 4.0.2 (R Core Team, 2013), and statistical significance was set at an alpha level of 0.05 after applying FDR correction to adjust for multiple comparisons.

### Data availability

The authors confirm that the data supporting the findings of this study are available within the article and its supplementary material. Raw data that support the findings of this study are available from the corresponding author upon reasonable request.

## Results

A total of 59 healthy controls, 41 patients with recent-onset schizophrenia, and 32 patients with chronic schizophrenia were included in the final analysis. The mean ages were 30.9 ± 5.2 years for healthy controls, 29.9 ± 5.4 years for patients with recent-onset schizophrenia, and 30.7 ± 5.5 years for patients with chronic schizophrenia. There were no significant group differences in age (*p* = 0.659) or sex (*p* = 0.627), confirming successful matching between the patient and control groups. As expected, significant group differences were observed in the FSIQ scores: 119.9 ± 9.2 for healthy controls, 100.7 ± 16.3 for recent-onset patients, and 83.0 ± 14.8 for chronic patients (*p* < 0.001). Among patients with schizophrenia, the mean illness durations were 2.6 ± 4.2 years for the recent-onset group and 10.9 ± 5.0 years for the chronic group. The mean antipsychotic dose was 16.9 ± 11.7 mg for the recent-onset group and 20.5 ± 14.4 mg for the chronic group, with no statistically significant difference between groups (*p* = 0.318). Patients with recent-onset schizophrenia exhibited significantly lower GAF scores (44.8 ± 14.5) than those with chronic schizophrenia (62.1 ± 11.0; *p* < 0.001). In contrast, the total PANSS scores did not differ significantly between recent-onset (62.6 ± 16.2) and chronic (68.3 ± 18.7) patients (*p* = 0.238). Detailed demographic and clinical information are presented in Table 1.

**Table 1.** Demographics and clinical characteristics of the study population

	Group			Statistic		
Variable	HC	Recent-onset SCZ	Chronic SCZ	F/t or chi	*p*	Post-hoc
Number of subjects	59	41	32			
Age, mean (SD), year	30.9 (5.2)	29.9 (5.4)	30.7 (5.5)	0.418	0.659	
Male, *n* (%)	19 (32.2)	17 (41.5)	11 (34.4)	0.933	0.627	
Duration of illness, mean (SD), year	NA	2.6 (4.2)	10.9 (5.0)			
Antipsychotic dose (mg)		16.9 (11.7)	20.5 (14.4)	−1.01	0.318	
PANSS positive, mean (SD)		15.8 (6.2)	15.6 (5.1)	−0.091	0.928	
PANSS negative, mean (SD)		17.9 (7.5)	18.2 (4.9)	0.176	0.861	
PANSS general, mean (SD)		28.9 (7.3)	34.4 (10.0)	2.349	0.023	
PANSS total, mean (SD)		62.6 (16.2)	68.3 (18.7)	1.195	0.238	
GAF, mean (SD)		44.8 (14.5)	62.1 (11.0)	4.367	< 0.001	
FSIQ, mean (SD)	119.9 (9.2)	100.7 (16.3)	83.0 (14.8)	33.9	< 0.001	H > R > C
MQ, mean (SD)	109.5 (12.7)	87.0 (19.9)	67.3 (14.1)	28.82	< 0.001	H > R > C

HC, healthy controls; SCZ: schizophrenia; H: healthy controls; SD: standard deviation; GAF, Global Assessment of Functioning; FSIQ, full-scale intelligence quotient; MQ: memory quotient; Antipsychotic dose is expressed as olanzapine-equivalent dose.

### Structural variability of cortical and subcortical measures

**Figure 1.**
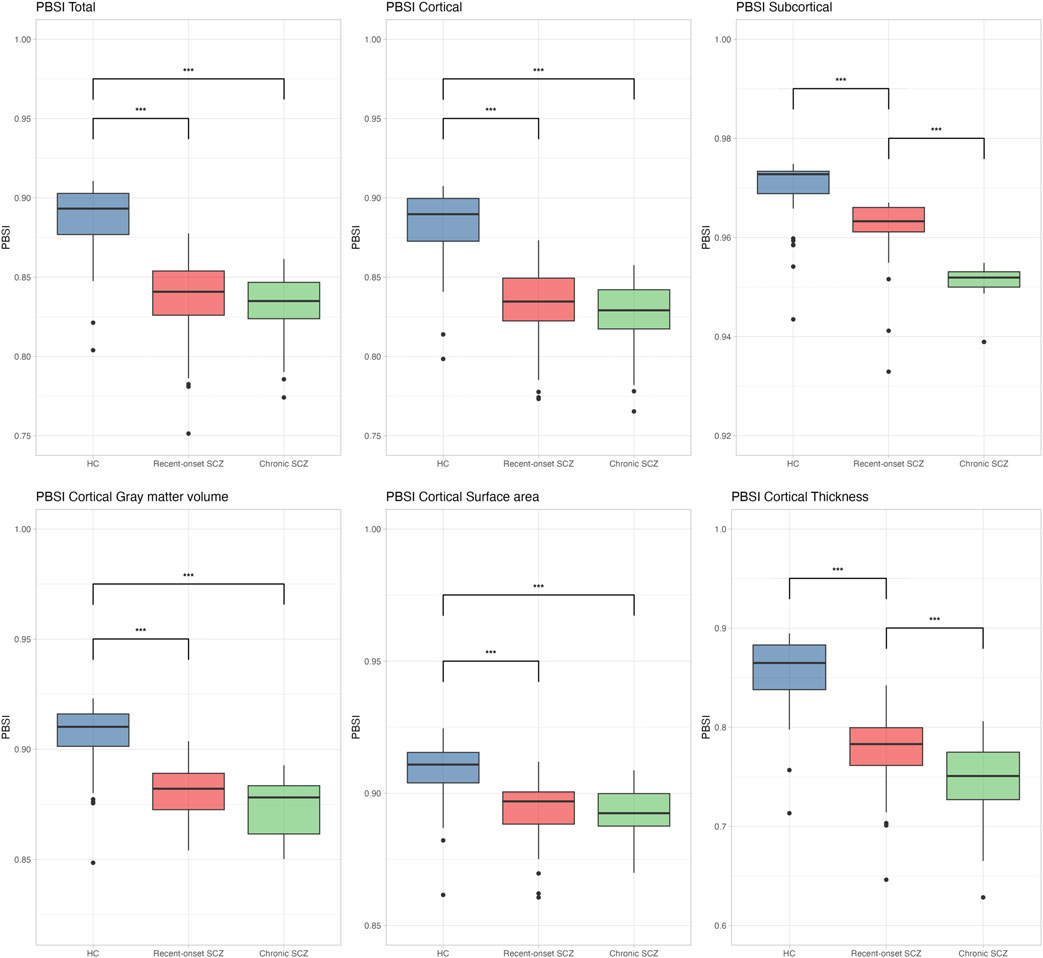
Group differences in PBSI scores. Boxplots illustrating group differences in PBSI scores among healthy controls (HC), patients with recent-onset schizophrenia (SCZ), and patients with chronic SCZ. Each plot is labelled with the corresponding PBSI measure in the top left corner. Statistical significance levels: *p < 0.05, **p < 0.01, and ***p < 0.001. PBSI: person-based similarity index.

The PBSI scores for all cortical and subcortical measures differed significantly between healthy controls, patients with recent-onset schizophrenia, and patients with chronic schizophrenia. Post-hoc analyses indicated that both patient groups exhibited significantly lower PBSI scores for all cortical and subcortical measures than healthy controls. Notably, significant differences between the recent-onset and chronic schizophrenia groups were observed only in the PBSI Subcortical and PBSI Thickness scores; the other PBSI scores did not differ significantly. Table 2 and Fig. 1 illustrate the group comparisons of the PBSI scores among patients with recent-onset schizophrenia, patients with chronic schizophrenia, and healthy controls.

**Table 2.** Group differences in PBSI scores

	HC		Recent-onset SCZ		Chronic SCZ		Statistic		Post-hoc
Variable	Mean	SD	Mean	SD	Mean	SD	*F*	*p*	
PBSI Total	0.887	0.022	0.837	0.028	0.831	0.022	75.285	< 0.001	H > R,C
PBSI Cortical	0.883	0.023	0.833	0.026	0.826	0.023	78.628	< 0.001	H > R,C
PBSI Subcortical	0.970	0.006	0.962	0.007	0.951	0.004	107.626	< 0.001	H > R > C
PBSI Grey matter volume	0.906	0.014	0.881	0.013	0.874	0.012	72.146	< 0.001	H > R,C
PBSI Surface area	0.908	0.012	0.893	0.012	0.892	0.010	27.965	< 0.001	H > R,C
PBSI Thickness	0.856	0.035	0.778	0.041	0.745	0.045	94.400	< 0.001	H > R > C

HC, healthy controls; SCZ, schizophrenia; H, healthy controls; SD, standard deviation; PBSI, Person-Based Similarity Index.

### Associations with clinical symptoms

In the recent-onset schizophrenia group, positive PANSS scores showed a significant negative correlation with the PBSI Surface area (*r* = −0.426, adjusted *p* = 0.03). Similarly, the PBSI Surface area was negatively correlated with both PANSS general scores (*r* = −0.421, adjusted *p* = 0.03) and PANSS total scores (*r* = −0.514, adjusted *p* = 0.013). However, no significant associations were found between the PBSI Surface area and PANSS negative scores or between any other PBSI and PANSS subscale scores. Furthermore, no significant correlations were found between the PBSI scores and cognitive measures (IQ and MQ) or GAF scores. In the chronic schizophrenia group, the PBSI scores did not correlate significantly with any clinical or cognitive measures. Detailed associations between the PBSI scores and clinical symptoms in patients with recent-onset and chronic schizophrenia are shown in Table 3 and Supplementary Table 2, respectively.

**Table 3.** Clinical correlations of PBSI scores in recent-onset schizophrenia

	PBSI Total		PBSI Cortical		PBSI Subcortical		PBSI Grey matter volume		PBSI Surface area		PBSI Thickness	
Variable	*r*	FDR p	*r*	FDR p	*r*	FDR p	*r*	FDR p	*r*	FDR p	*r*	FDR p
IQ	0.071	0.693	0.24	0.61	0.216	0.385	0.407	0.09	0.348	0.063	0.163	0.511
MQ	−0.139	0.532	0.104	0.61	0.403	0.154	0.055	0.765	0.09	0.626	0.24	0.511
PANSS positive	0.156	0.532	0.094	0.61	−0.065	0.714	−0.33	0.132	−0.426	0.03*	−0.066	0.714
PANSS negative	0.231	0.344	0.126	0.61	−0.161	0.506	−0.263	0.185	−0.346	0.063	−0.216	0.511
PANSS general	0.231	0.344	0.146	0.61	−0.317	0.237	−0.295	0.157	−0.421	0.03*	−0.137	0.523
PANSS total	0.271	0.344	0.162	0.61	−0.244	0.385	−0.382	0.09	−0.514	0.013*	−0.188	0.511
GAF	−0.322	0.344	−0.165	0.61	0.17	0.539	0.088	0.765	0.358	0.13	−0.251	0.511

PBSI, Person-Based Similarity Index; FDR, false discovery rate; IQ, intelligence quotient; MQ, memory quotient; GAF, Global Assessment of Functioning; *Statistically significant *p* < 0.05.

## Discussion

In this study, we investigated the structural variability in cortical and subcortical regions among patients with schizophrenia using neuroimaging data obtained from a single university-affiliated hospital. Using the PBSI scores to quantify structural brain variability, we identified significant differences among patients with recent-onset schizophrenia, those with chronic schizophrenia, and healthy controls. Post hoc analyses revealed significantly lower PBSI scores across all cortical and subcortical measures in patients with schizophrenia than in healthy controls, reflecting greater inter-individual heterogeneity in the brain structure. These findings align with those of previous studies that similarly reported reduced PBSI scores in patients with psychosis (Antoniades *et al*., 2021; Janssen *et al*., 2021; Omlor *et al*., 2025). The observed reduction in PBSI scores may indicate disrupted neurodevelopmental processes, such as abnormal synaptic pruning and impaired neural connectivity, which is consistent with findings from recent molecular imaging studies (Finnema *et al*., 2016). By replicating these findings, our study reinforces the neurodevelopmental model of schizophrenia.

Interestingly, a few significant differences emerged in the PBSI scores between the recent-onset and chronic schizophrenia groups. Although some evidence suggests neurocognitive deterioration as schizophrenia progresses, most PBSI scores did not differ significantly between the two patient groups. This finding might partly reflect the use of a five-year illness duration cut-off, which may inadequately capture subtle or continuous structural changes over the course of the disease. Additionally, it is plausible that structural heterogeneity (reflected by lower PBSI scores) emerges early in schizophrenia, potentially even before the onset of overt psychotic symptoms. This is consistent with previous research identifying early neurobiological impairments. Longitudinal studies are needed to determine whether structural brain variability remains stable or evolves with disease progression. Further analyses incorporating illness duration as a continuous variable could elucidate its impact on morphometric variability. Nonetheless, significant differences were observed between the chronic and recent-onset schizophrenia groups, specifically for cortical thickness and subcortical volume. These results are consistent with previous reports of progressive cortical thinning (van Haren *et al*., 2011; Zhao *et al*., 2022) and subcortical volume reduction (van Haren *et al*., 2016), particularly involving the putamen and pallidum (van Erp *et al*., 2016), following illness onset, suggesting that progressive structural changes may be region-specific rather than globally distributed.

Clinical correlations between the PBSI scores revealed mixed results. In the recent-onset schizophrenia group, the PBSI Surface area scores were negatively correlated with the PANSS-positive, general, and total scores, indicating that greater structural deviation may be associated with increased symptom severity during the early stages of schizophrenia. However, no significant associations emerged between the PBSI scores and cognitive measures (IQ or MQ), and no significant correlations were observed in the chronic schizophrenia group. These findings differ from those of our earlier study, in which lower PBSI scores were correlated with both higher symptom severity and cognitive impairment. Such discrepancies may arise from differences in sample size, given that separate analyses of the recent-onset and chronic groups reduced the statistical power and potentially introduced bias due to missing data. Additionally, the cognitive measures used in this study (IQ and MQ) may not have adequately captured executive dysfunction, a domain typically impaired in schizophrenia. Another potential confounder was antipsychotic medication exposure, which was not controlled in the present study. Medication exposure may influence the cortical and subcortical morphometry (Roiz-Santiáñez *et al*., 2012, Lesh *et al*., 2015; Krajner *et al*., 2022), which may have affected our results.

This study has several limitations that should be considered when interpreting the findings. First, we did not adjust for confounding factors, such as intelligence, education level, or socioeconomic status, all of which can affect cortical thickness (Menary *et al*., 2013; Piccolo *et al*., 2016; Habeck *et al*., 2020; Kang *et al*., 2020). These variables may have influenced the results. Second, our cognitive assessments were limited in scope and did not include comprehensive batteries, such as the Measurement and Treatment Research to Improve Cognition in Schizophrenia (MATRICS) Consensus Cognitive Battery (Nuechterlein *et al*., 2008), which could provide a more thorough evaluation of cognitive deficits in schizophrenia. Third, our recruitment strategy likely favoured patients with relatively mild or well-managed symptoms owing to the requirement for informed consent, introducing a sampling bias and potentially limiting the generalizability of our findings to more severely affected populations. Finally, the cross-sectional nature of our study prevents causal interpretations or definitive conclusions regarding disease progression. Moreover, the lack of control over antipsychotic medications prevents us from distinguishing the effects of medication from illness-related structural changes, particularly in chronic patients. However, there were no significant differences in antipsychotic dose between the recent-onset and chronic groups, suggesting that medication effects are unlikely to fully account for the observed group differences.

In summary, this study evaluated the morphometric variability in cortical and subcortical brain regions using neuroimaging data from 59 healthy controls, 41 patients with recent-onset schizophrenia, and 32 patients with chronic schizophrenia. As hypothesised, PBSI scores, which reflect the similarity of an individual’s structural profile to that of others in the same group, were significantly lower in patients with schizophrenia than in healthy controls across all cortical and subcortical measures. However, the differences between patients with recent-onset and chronic schizophrenia are modest, and the correlations between PBSI scores and clinical measures are limited. Nevertheless, these findings underscore the substantial interindividual heterogeneity in brain morphometry among patients with schizophrenia. Overall, this study provided valuable neurobiological insights into the clinical heterogeneity of schizophrenia.

## Supporting information

Jo et al. supplementary material 1 Jo et al. supplementary material

Jo et al. supplementary material 2 Jo et al. supplementary material

## Supplementary material

The supplementary material for this article can be found at https://doi.org/10.1017/neu.2025.10043.
