# Supplementary material for: Structural brain variability in recent-onset and chronic schizophrenia: evidence from person-based similarity index analysis
Source: Acta Neuropsychiatr. 2025 Nov 3;37:e89. doi: 10.1017/neu.2025.10043 (PMC13130360; doi:10.1017/neu.2025.10043)
Supplement: Jo et al. supplementary material 1 — Jo et al. supplementary material [file S0924270825100434sup001.docx]

**Supplementary Table 1.** Scanners and T1-weighted MRI parameters for the test samples

| Dataset | Scanners | Protocol parameters |
| --- | --- | --- |
| AMC 1 | 3T Philips Achieva | T1-weighted structural images were acquired with a 8-channel SENSE head-coil (TE = 4.6 ms, TR = 9.0 ms, voxel size = 1.0 × 1.0 × 1.0 mm, FOV = 240 × 240 × 170 mm, flip angle = 8°) |
| AMC 2 | 3T Philips Ingenia | TE = 4.6 ms, TR = 9.9 ms, voxel size = 1.0 × 1.0 × 1.0 mm, FOV = 240 × 240 × 170 mm, flip angle = 8° |
| AMC 3 | 3T Philips Ingenia CX | Sagittal T1-weighted structural images were acquired with a 32-channel dStream head coil and 3D, FFE sequence (TE = 2.9 ms, TR = 6.5 ms, flip angle = 9°, FOV (RL, AP, FH) = 211 × 256 × 256 mm, voxel size = 1.0 × 1.0 × 1.0 mm, 211 slices, slice thickness = 1 mm). |

FOV, field of view; MRI, magnetic resonance imaging.
