# Supplementary material for: Structural brain variability in recent-onset and chronic schizophrenia: evidence from person-based similarity index analysis
Source: Acta Neuropsychiatr. 2025 Nov 3;37:e89. doi: 10.1017/neu.2025.10043 (PMC13130360; doi:10.1017/neu.2025.10043)
Supplement: Jo et al. supplementary material 2 — Jo et al. supplementary material [file S0924270825100434sup002.docx]

**Supplementary Table 2.** Clinical correlations of PBSI scores in chronic schizophrenia

|  | PBSI Total | | PBSI Cortical | | PBSI Subcortical | | PBSI Gray matter volume | | PBSI Surface area | | PBSI Thickness | |
| --- | --- | --- | --- | --- | --- | --- | --- | --- | --- | --- | --- | --- |
| Variable | r | FDR p | r | FDR p | r | FDR p | r | FDR p | r | FDR p | r | FDR p |
| IQ | 0.357 | 0.131 | 0.359 | 0.128 | 0.327 | 0.377 | -0.079 | 0.865 | -0.345 | 0.534 | 0.429 | 0.225 |
| MQ | 0.416 | 0.119 | 0.419 | 0.115 | 0.372 | 0.377 | -0.136 | 0.865 | -0.042 | 0.863 | 0.356 | 0.225 |
| PANSS positive | -0.384 | 0.12 | -0.377 | 0.128 | -0.175 | 0.643 | 0.162 | 0.865 | 0.158 | 0.589 | -0.239 | 0.361 |
| PANSS negative | -0.451 | 0.109 | -0.445 | 0.11 | -0.325 | 0.377 | 0.014 | 0.954 | 0.21 | 0.534 | -0.414 | 0.225 |
| PANSS general | -0.439 | 0.109 | -0.438 | 0.11 | -0.098 | 0.683 | 0.082 | 0.865 | 0.207 | 0.534 | -0.326 | 0.225 |
| PANSS total | -0.46 | 0.109 | -0.456 | 0.11 | -0.188 | 0.643 | 0.093 | 0.865 | 0.209 | 0.534 | -0.347 | 0.225 |
| GAF | 0.275 | 0.227 | 0.272 | 0.232 | 0.142 | 0.643 | -0.177 | 0.865 | -0.231 | 0.534 | 0.188 | 0.428 |

PBSI, Person-Based Similarity Index; FDR: false discovery rate; IQ: intelligence quotient; MQ: memory quotient; GAF, Global Assessment of Functioning
